# Supplementary material for: Chiari II brain malformation is secondary to open spina bifida
Source: Dis Model Mech. 2026 Apr 24;19(4):dmm052528. doi: 10.1242/dmm.052528 (PMC13148473; doi:10.1242/dmm.052528)
Supplement: Supplementary information [file dmm-19-052528-s1.pdf]

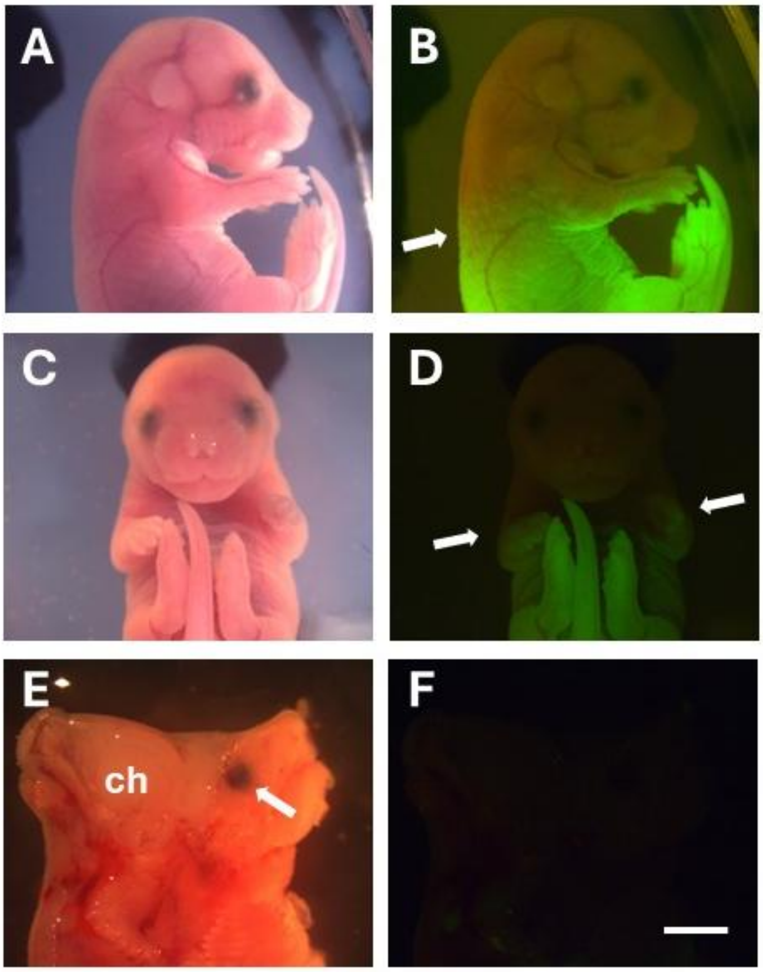

Fig. S1. Analysis of rostro-caudal level of *Cdx2^cre^* recombination in E18.5 fetuses. (**A-D**) Bright-field (A,C) and fluorescence (B,D) images of E18.5 *Cdx2$^{cre/+}$; Rosa26^{eYFP}* fetuses, viewed from the right side (A,B) and front (C,D). The cut-off level of *Cdx2^{cre}*-mediated eYFP recombination (arrows in B.D) is at the forelimb level, which corresponds to the thoraco-cervical boundary. (**E,F**) Fetal head bisected sagittally from the front (E; arrow indicates left eye; ch: cerebral hemisphere) to identify any evidence of brain Cdx2^{Cre} recombination, but none is seen in the fluorescence image (F). This analysis confirms our findings at E10.5 and E15.5 (Figures 1B,C; 2A-D) that *Cdx2^{cre}* mediates recombination in the trunk region only, and not in the head. Sample size = 3. Scale bar = 2 mm in F (for A-F).

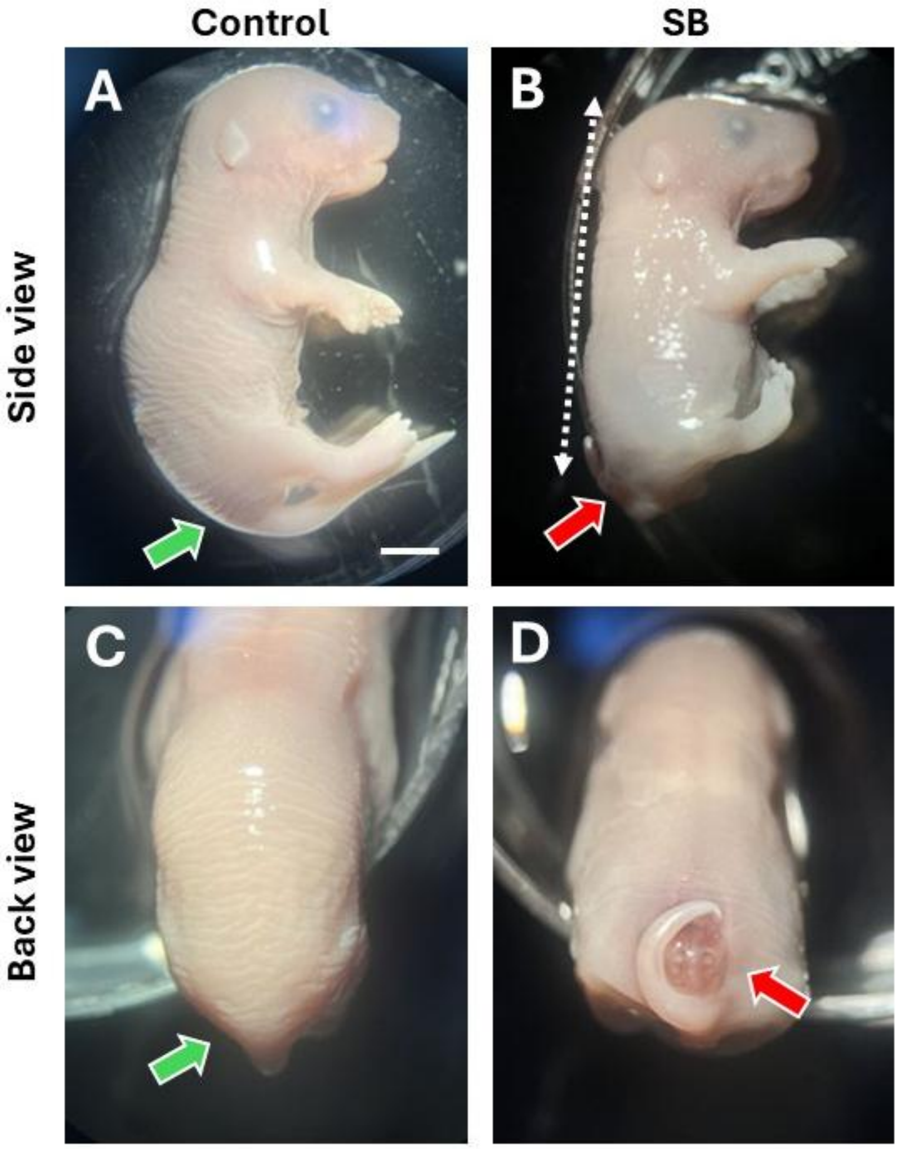

Fig. S2. Additional views of E18.5 control and SB fetuses. (A,C) Control and (B,D) SB fetuses shown from the right side (A,B) and back (C,D) views. Green arrows: normal low spinal region in control. Red arrows: open SB with a curled tail defect. Note the abnormally short body axis in the SB fetus (dotted arrow). Scale bar = 2 mm in all parts.

Disease Models & Mechanisms • Supplementary information

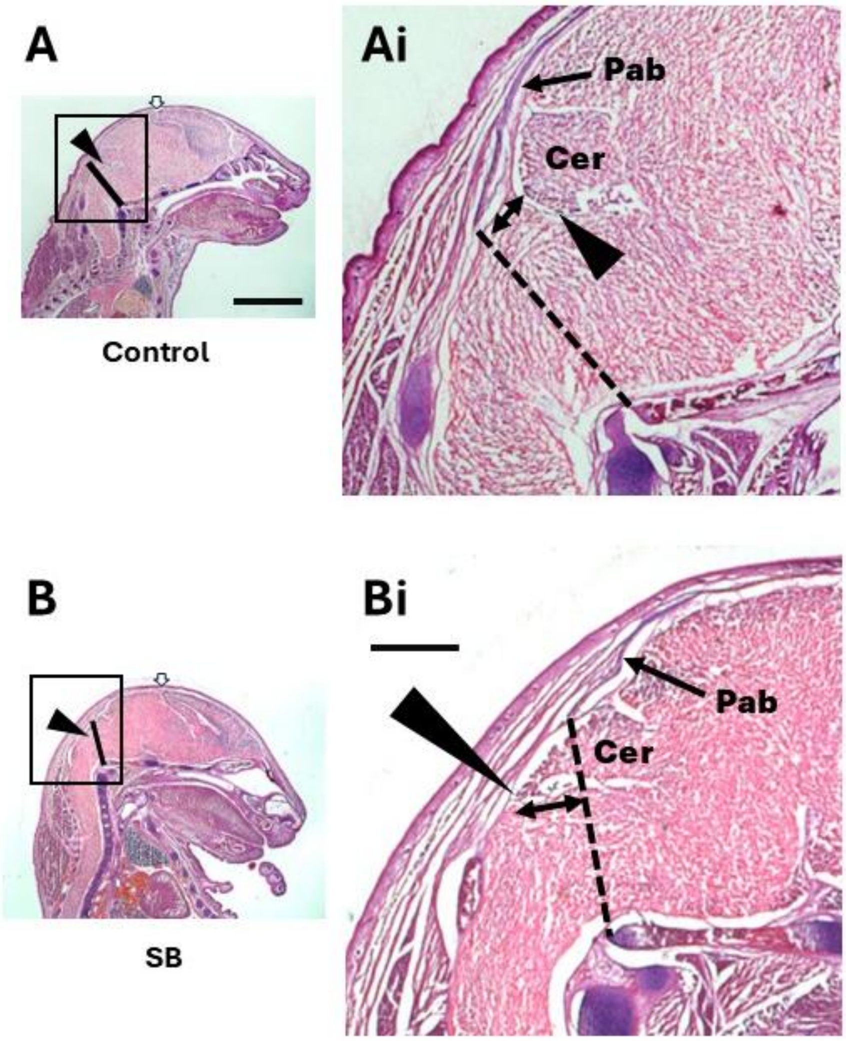

Fig. S3. Analysis of hindbrain herniation in SB fetuses, compared with controls at E18.5. Higher magnification views (Ai, Bi) of the H&E-stained sagittal head sections (A, B) as reproduced from Figure 3A, B. A line was drawn from the caudal edge of the parietal bone dorsally (Pab in Ai, Bi) to the inflection point of the skull base ventrally, where it joins the vertebral column (purple). This line (dashed in Ai, Bi) is taken as the boundary between skull and spinal region. In the SB head, the caudal edge of the cerebellum (Cer; black arrowhead in Bi) is located below the line (double headed arrow in Bi), indicating a herniation. In contrast, the control head shows the cerebellum entirely above/rostral to the line (double headed arrow in Ai) indicating no herniation. See Figure 3C, D for quantitation of skull boundary and cerebellar herniation. Parietal bone length was measured from its rostral edge (open arrows in A, B) to its caudal edge (Ai, Bi), and normalised to antero-posterior head length in each fetus. Normalised parietal bone length does not differ between control and SB (Student's t-test: p = 0.372; n = 3 for each genotype). Scale bars = 2 mm in A, B; 0.5 mm in Ai, Bi.

Disease Models & Mechanisms • Supplementary information

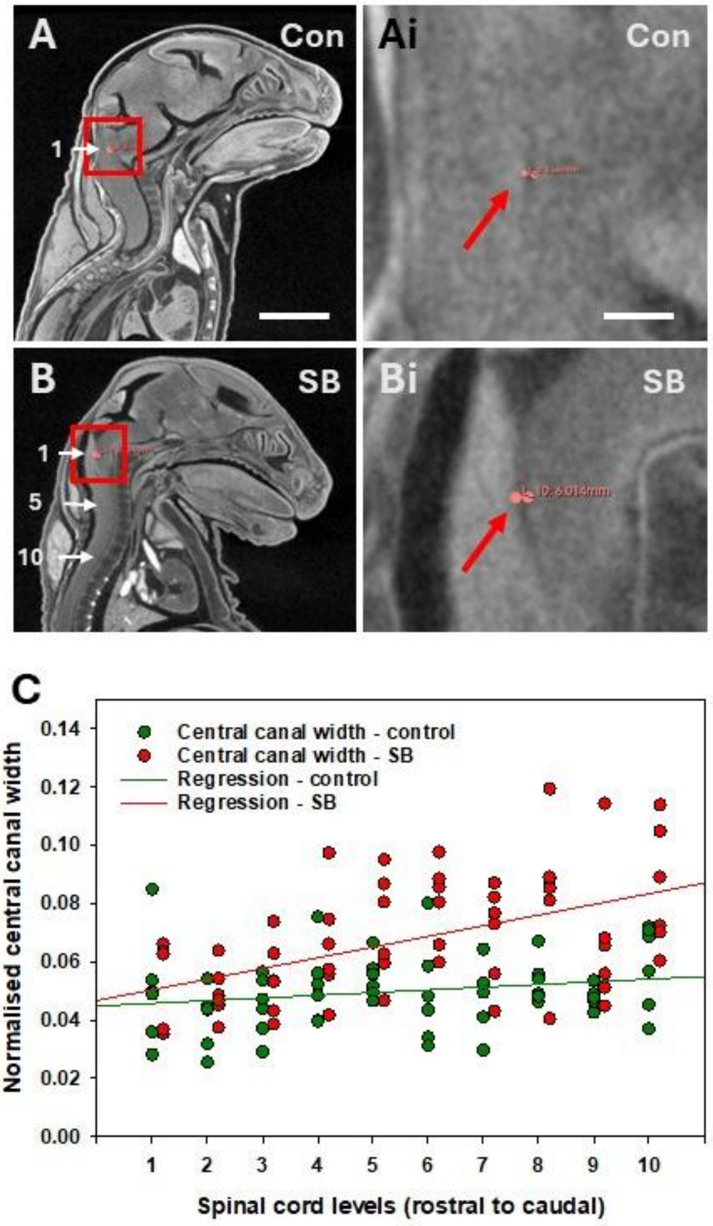

Fig. S4. Enlargement of the central spinal canal in SB fetuses. (**A-Bi**) Soft-tissue microCT images of control (Con; A, Ai) and SB (B, Bi) E18.5 fetal heads at low magnification (A, B) and with magnified views of the boxed areas (Ai, Bi). The central canal is visible as a dark vertical line against the grey spinal cord tissue in Ai, Bi (red arrows). Central canal width could be reproducibly measured in these sagittal images, and was determined at ten equally spaced levels, 1-10, moving down the spinal cord (numbered white arrows in A, B). Scale bars = 2 mm in A, B; 0.2 mm in Ai, Bi. (**C**) Central canal width,

Disease Models & Mechanisms • Supplementary information

normalised to spinal cord dorso-ventral width, plotted against spinal cord level in control and SB fetuses (n = 6 each). Statistical analysis: 2-way ANOVA shows significant differences between genotype (control vs SB) and spinal cord level ($p < 0.001$ for each). Linear regression equations: $Y = 0.0009 X + 0.04483$ (control) and $Y = 0.0037 X + 0.0467$ (SB). $R^2 = 0.30$ (control) and 0.71 (SB). Student t-tests show significant differences between regression line slopes ($p = 0.011$) but not Y-axis intercepts ($p = 0.762$).

Disease Models & Mechanisms • Supplementary information

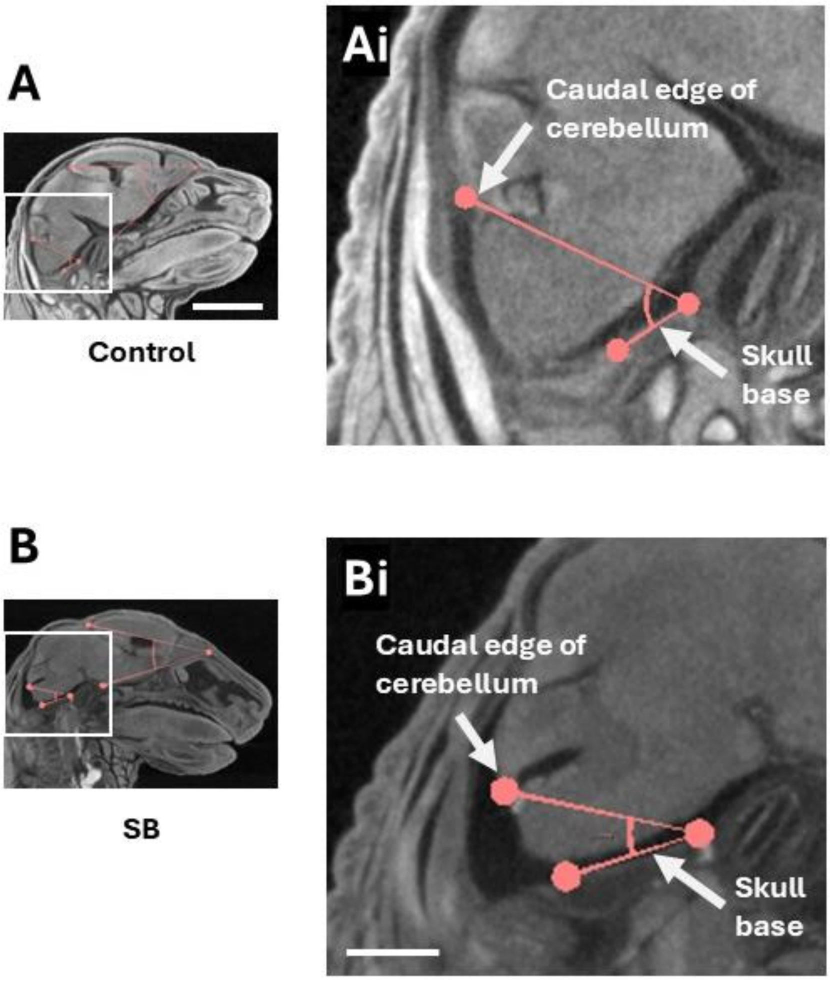

Fig. S5. Analysis of cerebellar position in relation to the skull base of SB and control fetuses at E18.5. Higher magnification views (Ai, Bi) are shown of the soft tissue microCT images (A, B) as reproduced from Figure 4K, L. Angle x (caudal edge of cerebellum to inflection point of the skull base) is smaller in the SB head (Bi) than in control (Ai), consistent with hindbrain herniation in the SB fetus. See Figure 4 N for quantitation. Scale bars = 2 mm in A, B; 0.5 mm in Ai, Bi.

Disease Models & Mechanisms • Supplementary information

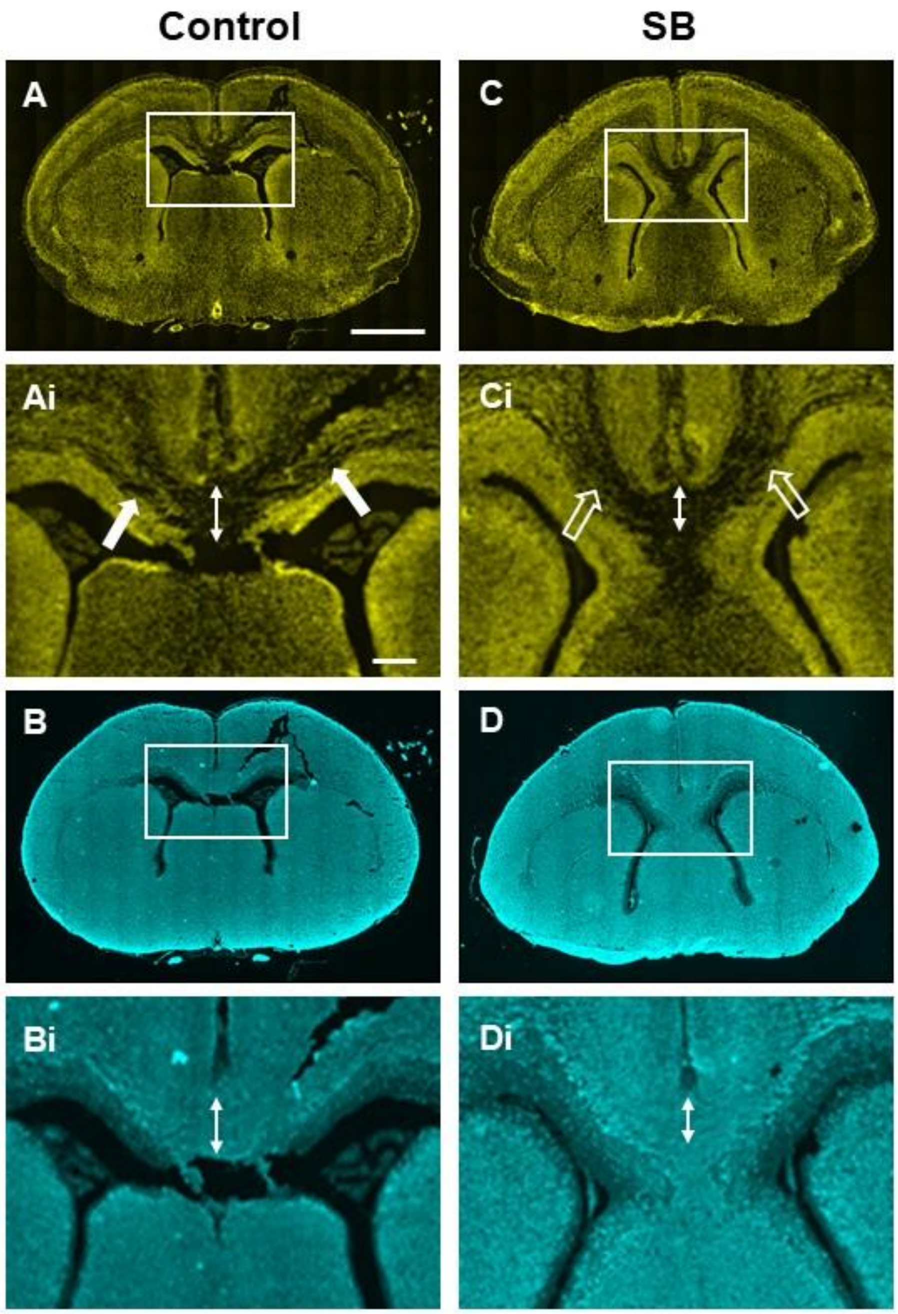

Fig. S6. Further example of callosal hypogenesis in an SB fetus at E18.5. Coronal sections through the brains of control (A, B) and SB (C, D) fetuses at low magnification, with boxed areas shown at higher magnification (Ai-Di). Sections stained with DAPI (A, Ai, C, Ci) and by Tuj1 immunohistochemistry (B, Bi, D, Di). Double headed arrows (Ai-Di) indicate the dorso-ventral thickness of the corpus callosum, which is reduced in the SB fetus (Bi, Di) compared with control (Ai, Ci). Note the abundant crossing fibres visible by DAPI staining that are entering the midline of the control fetus (solid arrows in Ai), which are greatly reduced in the SB fetus (open arrows in Bi). Scale bars = 1 mm in A -D; 0.2 mm in Ai-Di.

Disease Models & Mechanisms • Supplementary information

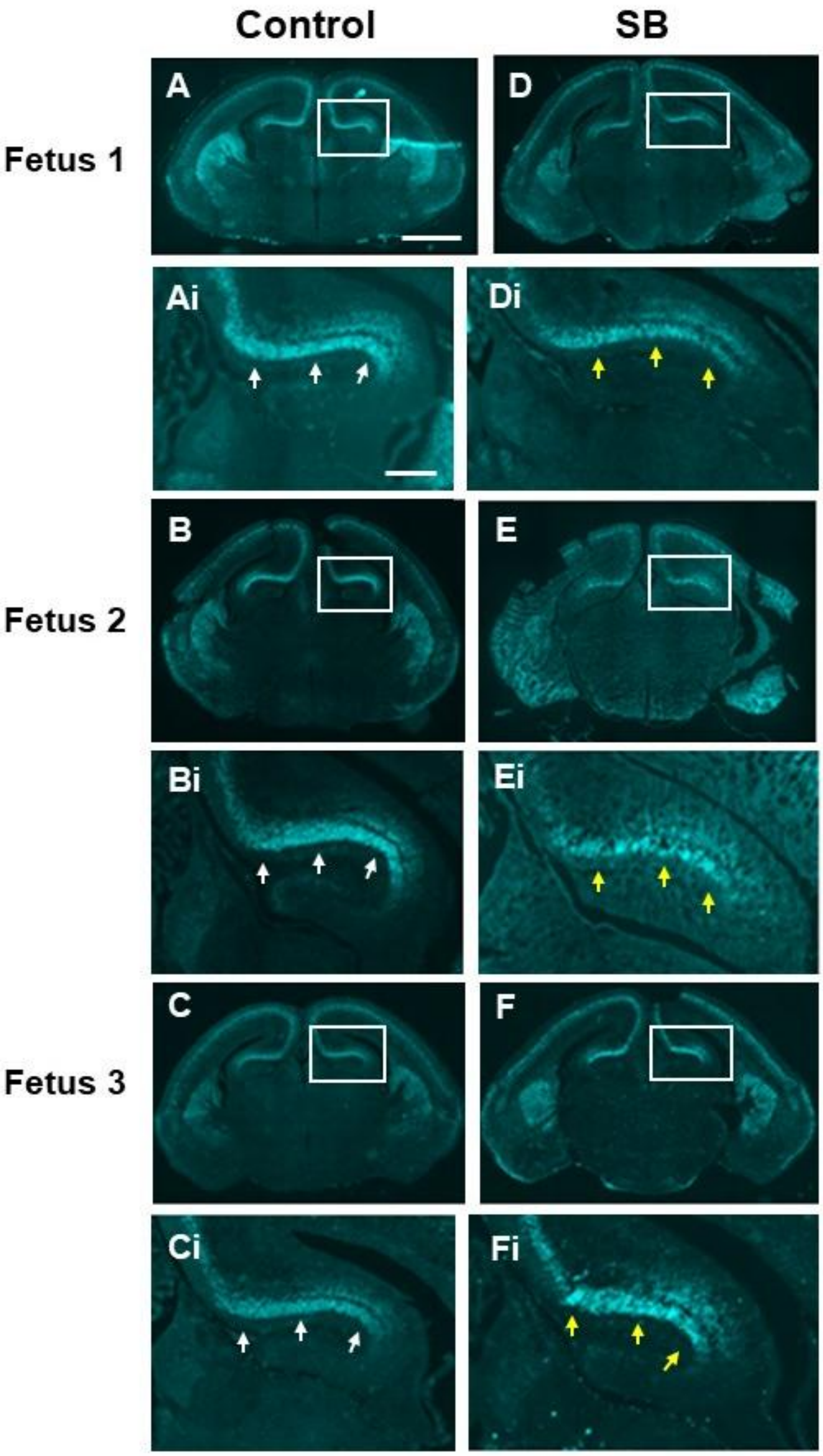

Fig. S7. Further examples of hippocampal hypogenesis in SB fetuses at E18.5. Coronal sections through the brains of three separate control (A-C) and SB (D-F) fetuses at low magnification, with boxed areas shown at higher magnification (Ai-Fi). Sections stained by CTIP2 immunohistochemistry. The CA1/CA2 regions of all three SB fetuses show reduced cellularity (yellow arrows in Di-Fi) compared with controls (white arrows in Ai-Ci). Control fetus 2 and SB fetus 1 are also shown (in adjacent sections) in Figure 7Q,R. Scale bars: 1 mm in A-F; 0.2 mm in Ai-Fi.

Disease Models & Mechanisms • Supplementary information

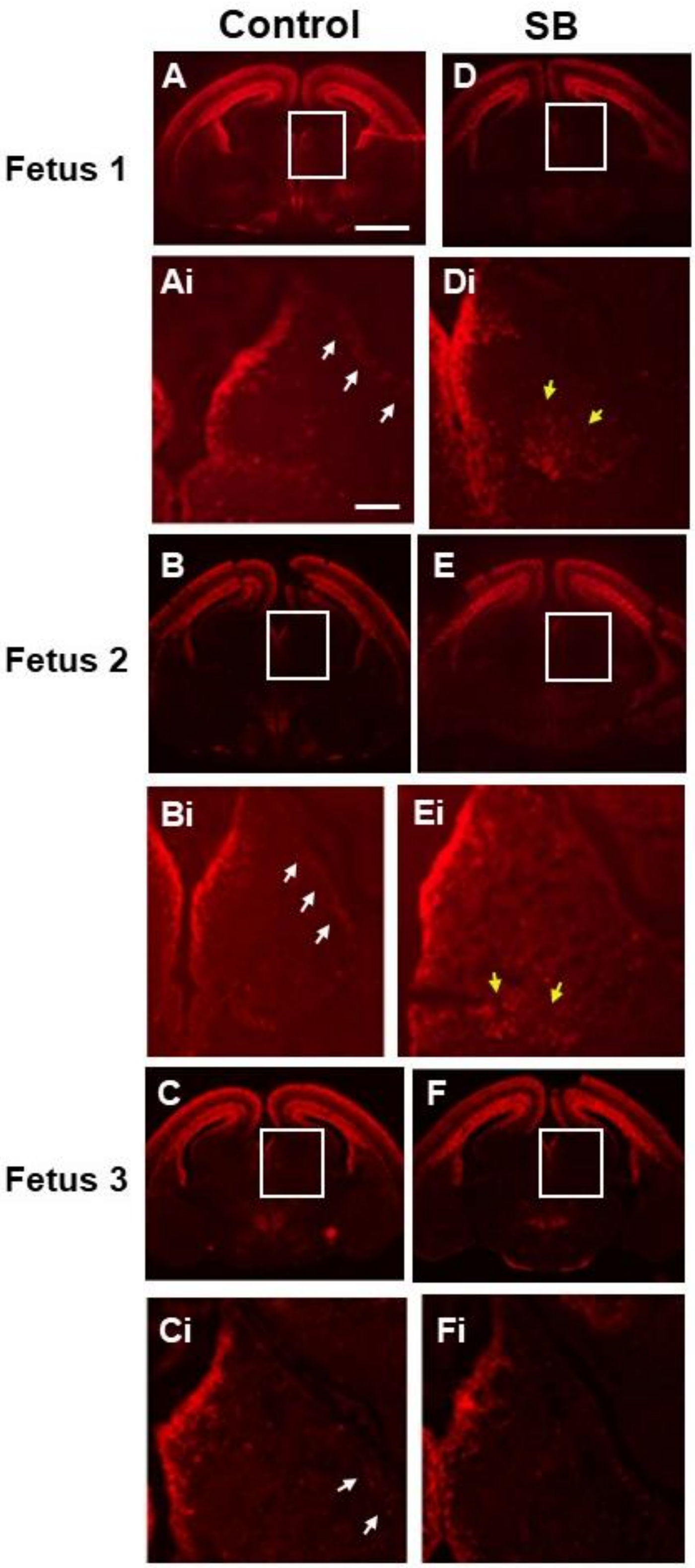

Fig. S8. Further examples of habenula hypogenesis in SB fetuses at E18.5. Coronal sections through the brains of three separate control (A-C) and SB (D-F) fetuses at low magnification, with boxed areas shown at higher magnification (Ai-Fi). Sections stained by BRN2 immunohistochemistry. White arrows indicate a slanting line of BRN2+ cells in the lateral habenula of two control brains (Ai, Bi), with a few cells visible at this location in the third control (Ci) brain. In contrast, such lateral habenular BRN2+ cells are not visible in any of the SB fetuses, although two show apparently increased BRN2+ cellularity in the ventromedial habenular region (yellow arrows in Di and Ei). Scale bars: 1 mm in A -F; 0.2 mm in Ai-Fi.

Disease Models & Mechanisms • Supplementary information

Table S1. Brain anomalies in human Chiari II

Brain region	Chiari II description
Cerebellar vermis & 4th ventricle	Downward displacement, peg-like shape [1]
Cisterna magna	Absent, producing “banana sign” [2]
Cerebellum	Folia shallow or absent [3]; heterotopia [4]
Tentorium cerebelli	Dysplastic [5]; low lying [6]
Cortex	Reduced thickness, heterotopia, smaller occipital surface areas [7, 8]
White matter	Total significantly reduced [8]
Ependyma	Denudation and rosettes [4]
Global neuronal nuclei	Varied heterotopia only present in SBA not in SBO [4]
Corpus callosum	Anomalies/partial agenesis [4, 9], hypoplasia (splenium and truncus affected most frequently) [10]
Pons	Hypoplastic [10]
Meninges	Dysplasia of subarachnoid space [4]
Ventricular system	Hydrocephalus, aqueductal stenosis [4]; ventriculomegaly [11]; enlarged lateral ventricles prenatally [9]
Mesencephalon	Hypoplastic [10]
Gyri	Stenogyria [4, 10, 12]; polymicrogyria [12]
Medulla	Medullary kinking [10]
Tectum	Tectal beaking [5, 11]
Colliculus	Collicular fusion [11]
Massa intermedia	Enlargement [13]
Habenular commissure	Elongation [13]
Pineal gland	Elongation [13]
Periventricular zone	Nodular heterotopia [14]
Cranial nerves	Hypoplasia [15]
Fetal proliferative zones (ventricular & subventricular)	Larger volumes [9]
Diencephalon (fetal)	Decreased volume [9]

References

1. Chiari, H. Ueber Veränderungen des Kleinhirns infolge von Hydrocephalie des Grosshirns. *Deutsche Medizinische Wochenschrift* 1891;17:1172-1175.

2. Van den Hof, M.C., Nicolaides, K.H., Campbell, J., Campbell, S. Evaluation of the lemon and banana signs in one hundred thirty fetuses with open spina bifida. *Am. J. Obstet. Gynecol* 1990;162:322-327.

3. Wolpert, S.M., Anderson, M., Scott, R.M., Kwan, E.S., Runge, V.M. Chiari II malformation: MR imaging evaluation. *AJR Am J Roentgenol* 1987;149:1033-1042.

4. Paschereit, F., Schindelmann, K.H., Hummel, M., Schneider, J., Stoltenburg-Didinger, G., Kaindl, A.M. Cerebral Abnormalities in Spina Bifida: A Neuropathological Study. *Pediatr Dev Pathol* 2022;25:107-123.

5. Peach, B. Arnold-Chiari Malformation: Anatomic Features of 20 Cases. *Arch Neurol* 1965;12:613-621.

Disease Models & Mechanisms • Supplementary information

6. Gardner, W.J. *The Dysraphic States. From Syringomyelia to Anencephaly*. Excerpta Medica, Amsterdam; 1973.

7. Gilbert, J.N., Jones, K.L., Rorke, L.B., Chernoff, G.F., James, H.E. Central nervous system anomalies associated with meningomyelocele, hydrocephalus, and the Arnold-Chiari malformation: Reappraisal of theories regarding the pathogenesis of posterior neural tube closure defects. *Neurosurgery* 1986;18:559-564.

8. Juranek, J., Fletcher, J.M., Hasan, K.M., et al. Neocortical reorganization in spina bifida. *Neuroimage* 2008;40:1516-1522.

9. Masse, O., Kraft, E., Ahmad, E., et al. Abnormal prenatal brain development in Chiari II malformation. *Front Neuroanat* 2023;17:1116948.

10. Schneider, J., Mohr, N., Aliatakis, N., et al. Brain malformations and cognitive performance in spina bifida. *Developmental Medicine and Child Neurology* 2021;63:295-302.

11. Nagaraj, U.D., Bierbrauer, K.S., Zhang, B., Peiro, J.L., Kline-Fath, B.M. Hindbrain herniation in Chiari II malformation on fetal and postnatal MRI. *AJNR Am. J Neuroradiol* 2017;38:1031-1036.

12. Bekiesinska-Figatowska, M., Duczkowska, A., Bragoszewska, H., Duczkowski, M., Mierzewska, H. Stenogyria - not only in Chiari II malformation. *J Neurol Sci* 2014;347:337-340.

13. Gooding, C.A., Carter, A., Hoare, R.D. New ventriculographic aspects of the Arnold-Chiari malformation. *Radiology* 1967;89:626-632.

14. Hino-Shishikura, A., Niwa, T., Aida, N., Okabe, T., Nagaoka, T., Shibasaki, J. Periventricular nodular heterotopia is related to severity of the hindbrain deformity in Chiari II malformation. *Pediatr. Radiol* 2012;42:1212-1217.

15. Tubbs, R.S., Oakes, W.J. *The Chiari Malformations*. Springer Science & Business Media, Google e-book; 2013.
